# An International External Quality Assessment Scheme to Assess the Diagnostic Performance of Polymerase Chain Reaction Detection of *Acanthamoeba* Keratitis

**DOI:** 10.1097/ICO.0000000000003275

**Published:** 2023-05-04

**Authors:** Maarten J. Sarink, Rob Koelewijn, Foekje Stelma, Titia Kortbeek, Lisette van Lieshout, Pieter W. Smit, Aloysius G. M. Tielens, Jaap J. van Hellemond

**Affiliations:** * Department of Medical Microbiology and Infectious Diseases, Erasmus MC University Medical Center, Rotterdam, the Netherlands;; † Department of Medical Microbiology, Radboudumc Nijmegen, the Netherlands;; ‡ National Institute of Public Health and the Environment, RIVM, Bilthoven, the Netherlands;; § Department of Parasitology, Leiden University Medical Centre, Leiden, the Netherlands; and; ¶ Department of Medical Microbiology, Molecular Diagnostics Unit, Maasstad Hospital, Rotterdam, the Netherlands.

**Keywords:** *Acanthamoeba*, diagnosis, nucleic acid amplification techniques, *Acanthamoeba* keratitis, external quality assessment

## Abstract

The purpose of this study was to assess the variation in methods and to determine whether an External Quality Assessment Scheme (EQAS) for polymerase chain reaction (PCR) detection of *Acanthamoeba* keratitis is valuable for the diagnostic process.

**Methods:**

A multicenter EQAS was introduced, covering 16 diagnostic laboratories. Using *Acanthamoeba castellanii* ATCC strain 30010, 3 sets of samples were prepared, containing different amounts of DNA, cysts, or trophozoites. Samples were masked and sent to the participants with instructions for use and a questionnaire concerning the applied methodologies. Special attention in this questionnaire was given to the used pretreatment methods to assess existing variations in these procedures.

**Results:**

A large variation in the methodologies and substantial differences in the diagnostic performance were found between participants. In contrast to the DNA samples where all participants had a perfect score, several false negative results were reported for the samples containing cysts or trophozoites. Only 9 participants had an optimal score, whereas one participant reported all samples as negative, one participant reported failures due to inhibition, and the other 5 reported in total 7 false negative results. A clear correlation was noticed between the PCR detection rate and the number of cysts or trophozoites in the sample.

**Conclusions:**

The results indicate that a pretreatment procedure can be a risky step in PCR-based detections of *Acanthamoeba*, but it improves the sensitivity and reliability, especially of samples containing cysts. Therefore, participation in an EQAS is informative for routine diagnostic laboratories and can assist in improving the laboratory procedures used for the diagnosis of *Acanthamoeba* keratitis.

## Abstract 2

Supplemental Digital Content is Available in the Text.

*Acanthamoeba* is a free-living amoeba that is ubiquitously present around the world in soil and water. It can exist in a trophozoite stage, which is actively moving and feeding, and a cyst stage, which is dormant and stress-resistant. *Acanthamoeba* can cause an infectious keratitis, which leads to blindness if left untreated or is treated with the wrong medication. *Acanthamoeba* keratitis is often seen in contact lens wearers, among which 1 in 21,000 in the Netherlands is affected.^1^ In recent years, this incidence has been reported as rising.^1–3^

The most important factor that determines the prognosis of *Acanthamoeba* keratitis is an early diagnosis.^4^ However, diagnosing *Acanthamoeba* keratitis requires clinical expertise, as symptoms overlap with infectious keratitis caused by other micro-organisms.^5^ Many different diagnostic tools are available, all with different characteristics. Direct culture is a laborious and time-consuming diagnostic method, in which the clinical sample is added to a non-nutrient agar plate seeded with Gram-negative bacteria (eg *Escherichia coli*), after which *Acanthamoeba* growth must be detected microscopically. Direct visualization of *Acanthamoeba* on the infected eye is also possible using in vivo confocal microscopy, but this method requires specialized equipment and personnel.^5^ Nucleic acid amplification tests (NAATs), such as polymerase chain reaction (PCR), have also been developed to detect *Acanthamoeba* DNA. These NAAT methods have a high sensitivity and can provide rapid results in contrast to the time-consuming culture procedures.^6,7^ However, a large diversity of NAATs are in use, and so far, external quality assessment scheme (EQAS) for these tests is lacking. An EQAS involves the comparison of test results of a laboratory to a source outside of that laboratory, allowing an objective assessment of the diagnostic performance of a laboratory.^8^ EQAS has already been used to optimize the detection and evaluation of other infectious diseases using NAATs.^9,10^

Here, we describe the evaluation of the introduction of an EQAS for the detection of *Acanthamoeba* cysts and trophozoites by NAAT. We aimed to assess the variation in methods and to determine whether an EQAS for the detection of *Acanthamoeba* can be of value for the diagnostic process.

## MATERIALS AND METHODS

### Sample Preparation

*Acanthamoeba castellanii* ATCC strain 30010 (“Neff”) was grown in cell culture flasks at 25°C in PYG medium, which contained proteose peptone, yeast extract, glucose, salt additives (ATCC medium 712), 40 µg/mL gentamicin, 100 units/mL penicillin, and 100 µg/mL streptomycin, as described before.^11^ To prepare samples containing trophozoites, cultures were refreshed with PYG on the day before sampling to ensure that only trophozoites were obtained. Trophozoites were collected by placing a cell culture flask on ice for 20 minutes and repeatedly tapping to detach trophozoites. To prepare samples with cysts, encystation was induced by replacing the supernatant of a culture of trophozoites growing in logarithmic phase with encystation medium, containing 95 mM NaCl, 5 mM KCl, 8 mM MgSO_4_, 1 mM NaHCO_3_, 0.4 mM CaCl_2_, and 20 mM Tris-HCl (pH 9.0), as described before.^12^ Cysts were collected by repeatedly tapping and pouring contents out of the cell culture flask. After collection, the trophozoite and the cyst suspensions were washed three times with Tris NaCl (25 mM Tris, 120 mM NaCl, pH 7.4) or encystation medium, respectively. Amoebae were then counted three times using a Bürker cell counter, after which the average count was used to prepare distinct dilutions of the amoebae, cysts in encystation medium, and trophozoites in TRIS NaCl. EDTA (5 mM) was added to the trophozoite suspensions to stop trophozoite replication and block DNA degradation. Finally, suspensions of 2000, 200, 20, or 2 amoebae in 200 µL medium were put in screw-cap Eppendorf tubes. Negative controls containing only TRIS NaCl + EDTA or encystation medium were also prepared. Purified *Acanthamoeba* DNA samples were prepared from a dense trophozoite culture, from which DNA was isolated by the MagNA Pure system (Roche, Basel, Switzerland). The isolated DNA was diluted in Tris-HCl (10 mM) + EDTA (0.5 mM) buffer (pH 8.0) supplemented with 10 mg/L Sheared Salmon DNA (Thermo Fisher, Waltham, MA) and 10 mg/L bovine serum albumin (Roche, Basel, Switzerland) to obtain DNA samples with a high, medium, or low concentration of *Acanthamoeba* DNA, comparable with the DNA content of the samples with 2000, 200, and 20 amoebae, respectively. A volume of 50 µL of each dilution was added to screw-cap Eppendorf tubes, along with a negative control containing only Tris-HCl + EDTA buffer with supplements.

### Sample Validation

The homogeneity of the prepared samples was validated by an expert laboratory by examining 5 randomly chosen replicates. The stability of the samples was tested by 2 expert laboratories by examining the samples directly after preparation as well as after storage at room temperature for 4 months when the EQAS distribution had been completed, and all results by all participants had been reported.

In this pilot study, 16 laboratories participated in the *Acanthamoeba* External Molecular Quality Assessment Scheme organized by the parasitology section of the Dutch Foundation for Quality Assessment in Medical Laboratories (SKML). All participating laboratories are accredited large hospital microbiological laboratories or national institutes of disease control. The samples were sent in a masked fashion to the participating laboratories, along with instructions for use and a questionnaire (see Appendix, Supplemental Digital Content, http://links.lww.com/ICO/B547).

## RESULTS

### Validation of the Homogeneity and Stability of the Samples

After preparation, the homogeneity of the samples was verified in an expert laboratory by a five-fold examination using DNA extraction followed by real-time PCR (rtPCR) analysis. The samples containing 20, 200, or 2000 cysts or trophozoites were positive in all these analyses and formed the set of validated samples. For these validated samples, the average standard deviation for all separate sample types was less than 1.5 cycles (Supplemental Table S1, Supplemental Digital Content, http://links.lww.com/ICO/B550). Of the samples that according to the dilution series should contain 2 cysts or 2 trophozoites, a positive result was found for only 3 of the 5 cyst samples and 1 of the 5 trophozoite samples. It is possible that due to the intrinsic unreliability of diluting suspensions, some of these samples that were supposed to contain 2 amoebae, contained in fact no amoebae. Another possibility is that the amount of DNA isolated from these samples was below the limit of detection. A combination of both possibilities is conceivable. The samples with 2 amoebae were included for educational purposes in the shipment to the participants, but the reported results of these samples were not used in our evaluation.

To test the stability of the samples, 2 expert laboratories analyzed them at the time of distribution and retested them circa 4 months later when the distributed samples had been analyzed and reported by all participants. In the period between preparation and retesting, the samples were stored at room temperature, similar to the distributed samples. These examinations revealed an average difference per sample of fewer than 1.5 cycles (Supplemental Table S2, Supplemental Digital Content, http://links.lww.com/ICO/B551) for all sample types (purified DNA, cysts, and trophozoites), demonstrating the stability of all types of samples over the entire study period.

### Variations in Methodology Among the Participants

**FIGURE 1.**
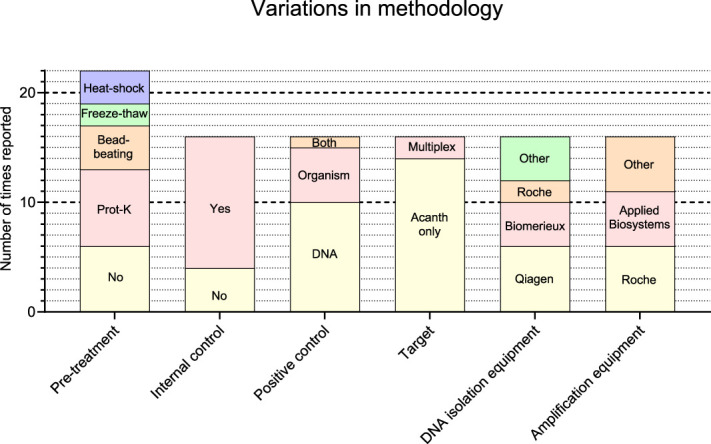
Overview of the used methodologies as reported by the 16 participating laboratories. Several participants used a combination of pretreatment procedures, which results in a total of reported pretreatment procedures of over 16. The combinations that reported were the following: Prot-K + heat–shock: 3 times, Prot-K + freeze–thaw: 2 times, and Prot-K + bead-beating: 1 time. “Multiplex” indicates that apart from *A. castellanii,* the amoebae *Balamuthia mandrillaris* and *Naegleria fowleri* were targeted.

The 16 laboratories that participated in this EQAS did so by reporting results and by completing the online questionnaire (see Appendix, Supplemental Digital Content, http://links.lww.com/ICO/B547), which aimed to provide an overview of the used methodology. Figure 1 shows a summary of the different methods that were used. Before performing DNA extraction by a commercial kit or platform, many participants used a specific pretreatment to enhance lysis of the amoebae by proteinase K treatment (n = 7/16), bead-beating (n = 4/16), heat–shock (n = 3/16), or freeze–thawing (n = 2/16). Some participants used a combination of these methods; one participant reported pretreatment as classified and 6 participants did not use any specific treatment before DNA extraction.

The equipment used for DNA extraction originated from several companies, among which Qiagen, Biomerieux, and Roche (Fig. 1). All laboratories used the whole sample volume (200 μL) for DNA extraction. The volume in which the extracted DNA was eluted differed substantially, ranging from 12.5 to 200 μL, with an average of 90.2 μL (median 100 μL). The volume of isolated DNA that was used in the PCR reaction also differed substantially, ranging from 2 to 20 μL (average 6.3 μL, median 5 μL). Laboratories used different equipment for DNA amplification, among which Roche (n = 6), Applied Biosystems (n = 5) and other (n = 5). Four of 16 participants did not include an internal control in their examination protocol, whereas all participants used a positive control, which was a DNA standard (10/16), an *Acanthamoeba* culture (5/16), or both (1/16). Two participants used a multiplex PCR in which also *Balamuthia mandrillaris* and *Naegleria fowleri* were targeted, whereas the other 14 participants used a PCR in which only *Acanthamoeba* spp. was examined.

### Qualitative Results

**FIGURE 2.**
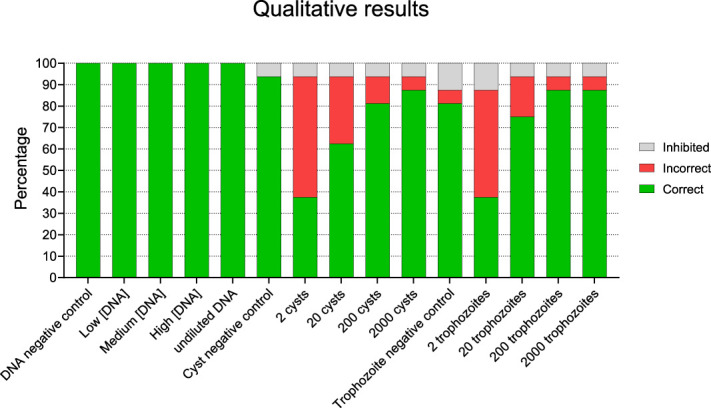
Qualitative results of samples containing different numbers of either cysts or trophozoites. All 16 participants reported their results for all samples, and these are all included in the figure.

All 16 participating laboratories reported qualitative results of DNA amplification tests of samples containing *A. castellanii* DNA, cysts, trophozoites, and respective negative controls. All 16 laboratories reported a positive result for all the samples containing purified *A. castellanii* DNA. By contrast, for the samples containing cysts or trophozoites for which DNA had to be isolated first, not all samples were reported to contain amoebae. The reported results of these samples containing cysts or trophozoites clearly correlated with the number of organisms that were present in the sample (Fig. 2). Ten of the 16 participants reported a positive result for at least 1 of the 2 educational samples, each containing 2 cysts or 2 trophozoites, and 2 of those 10 participants reported positive results for both samples (Supplemental Table S3-A, Supplemental Digital Content, http://links.lww.com/ICO/B552). The sample with 20 cysts or 20 trophozoites was reported as positive by 10 and 12 participants, respectively. Nine of the 10 participants who reported a positive result for the cyst sample reported a positive result for the trophozoite sample as well. The sample with 200 cysts or 200 trophozoites was reported as positive by 13 and 14 participants, respectively. For the samples containing 20 *Acanthamoeba* cysts or trophozoites, possible aliquoting errors cannot explain the lower detection rate than observed for the samples containing 200 amoebae because the validation by 5-fold analysis by a single laboratory showed only small quantitative differences in these samples.

The incorrect results were not evenly distributed among participants, as one participant reported inhibition for all samples containing amoebae and another participant reported negative results for those samples (Supplemental Table S3-A, Supplemental Digital Content, http://links.lww.com/ICO/B552). Altogether, for the 6 validated amoeba samples, 9 participants reported all correct qualitative results, 3 reported a single false negative result, 2 reported 2 false negative results, and as mentioned earlier, one participant reported 6 false negative results, and another one reported 6 failures of examination due to inhibition of the DNA replication (Supplemental Fig. S1, Supplemental Digital Content, http://links.lww.com/ICO/B548 and Supplemental Table S3-B, Supplemental Digital Content, http://links.lww.com/ICO/B552).

### Quantitative Results

All participants used an rtPCR method for the DNA amplification and reported their results also in a semiquantitative manner by providing the Cq-values, which indicate the PCR cycle at which the fluorescent signal of the rtPCR reactions exceeded the threshold for background fluorescence. A high Cq-value indicates a low quantitative load of amoebae, whereas a low Cq-value indicates a higher quantitative load of amoebae. Supplemental Digital Content (Supplemental Table S3, http://links.lww.com/ICO/B552) contains all reported Cq-values and the calculations used in our evaluations. Rather large differences between the participants were reported in the Cq-values of the DNA samples. However, the amplification of the 3 DNA samples resulted for all participants in a regular, stepwise decrease in Cq-values with increasing DNA content from low through medium to high. This means that although differences exist between the participants in the efficiency of the PCR reaction, within each laboratory, the results of the analyses of the 3 samples with different DNA content are consistent (Supplemental Fig. S1A, Supplemental Digital Content, http://links.lww.com/ICO/B548).

The results of the analyses of the validated amoeba samples were not as good as the ones of the DNA samples, although the 2 laboratories that reported inhibition or negative results for all amoeba samples were not included in this further evaluation. As mentioned, 5 of the 14 remaining laboratories reported in total 7 false negative results for the validated amoeba samples (Supplemental Fig. S1 B-C, Supplemental Digital Content, http://links.lww.com/ICO/B548 and Supplemental Table S3-B, Supplemental Digital Content, http://links.lww.com/ICO/B552). Furthermore, in sharp contrast to the stepwise decrease in Cq-values with an increasing amount of DNA that was reported by all laboratories for the set of the 3 different DNA samples, in more than half of the laboratories, the analyses of the sets of 20, 200, and 2000 cysts or trophozoites did not result in this predictable pattern (Supplemental Fig. S1 B-C, Supplemental Digital Content, http://links.lww.com/ICO/B548). Merely 5 laboratories reported such a consistent pattern for both the cyst and the trophozoite samples.

**FIGURE 3.**
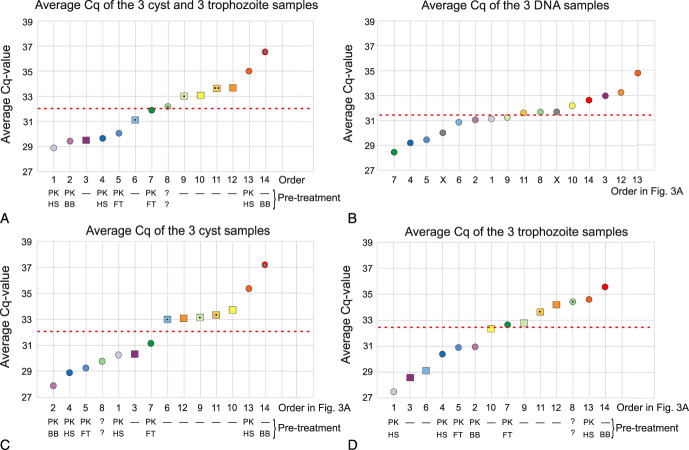
Variation in the Cq-values as reported by the participants for the various sample types. The participants were sorted in ascending order (1→14) based on their average Cq-value of the 3 cyst plus the 3 trophozoite samples (panel A). Square symbols indicate that the participant did not use a pretreatment in the analysis of the amoeba samples. Circles indicate that the participant used a pretreatment in the analyses of the amoeba samples, and this pretreatment is presented underneath the graph. In the top line beneath each graph is shown whether proteinase K was used as pretreatment (PK); in the bottom line beneath each graph is shown whether (also) another pretreatment was used, BB, bead beating; HS, heat–shock; FT, freeze–thaw cycle; ?, unknown pretreatment. Panels B, C, and D show in the same way the average Cq-values of the DNA, the cyst, and the trophozoite samples, respectively. Each black dot in the circles and squares indicates a false negative result in the samples of that graph. To facilitate a comparison of the results of the participants in the various sample types, underneath graphs B, C, and D is the order number shown of that participant in the spectral sorting from violet (1) to red (14) in panel A. For example, the participant with the lowest average Cq-value for the 3 DNA samples (green circle, utmost left in panel B) was sorted as #7 in panel A. The individual indicator color of the circle or square is for each participant the same in the 4 graphs. The median Cq-value is indicated in each graph (A–D) with a dashed red line. For details and calculations, see Supplemental Table S3 (Supplemental Digital Content, http://links.lww.com/ICO/B552).

Details of the variation in the results of the participating laboratories were further investigated. The entire analytical procedure for amoeba samples consists of several steps: a pretreatment if applied, the DNA isolation, and the rtPCR method. As a measure of the efficiency of this entire analytical procedure, the average of the reported Cq-values of the 3 cyst samples plus the 3 trophozoite samples was calculated for each participant. False negative results were assigned a Cq-value of 39.5, as this was the highest Cq-value reported by any participant for these amoeba samples. After this calculation, the participants were sorted in ascending order (1→14), based on this combined performance in the 6 samples containing amoebae (Fig. 3A and Supplemental Table S3-C, Supplemental Digital Content, http://links.lww.com/ICO/B552). The average Cq-values of the sets of DNA, cyst, and trophozoite samples are shown in Figure 3 in panels B, C, and D, respectively (Supplemental Table S3-D, Supplemental Digital Content, http://links.lww.com/ICO/B552). The difference in the average Cq-value between the most efficient analysis (lowest Cq-value) and the least efficient one (highest Cq-value) was smaller for the set of DNA samples (6.3) than for the sets of cyst (9.3) and trophozoite (8.1) samples (Fig. 3 B, C, D; Supplemental Table S3-D, Supplemental Digital Content, http://links.lww.com/ICO/B552). In addition, the spreading around the median Cq-value was much higher in the amoeba samples than in the DNA samples. Fifty percent (8/16) of the analyses of the DNA samples were within the range of plus 1 Cq-value to minus 1 Cq-value of the median Cq-value, whereas 14% (2/14) of the cysts analyses and 21% (3/14) of the trophozoite analyses where within that range (Fig. 3 and Supplemental Table S3-E, Supplemental Digital Content, http://links.lww.com/ICO/B552).

### Relationship Between Methodology and Reported Low or High Cq-Values

The participants reported the details of their procedure which enabled the calculation of the percentage of the amount of DNA isolated from the sample that was used in the PCR. As all laboratories used the entire sample (200 μL) for the extraction of DNA, this is also the percentage of the original sample used in the PCR reaction. This ranged from 1.5% to 16.7% with an average of 7.6% (median 8.2%). This percentage of the original sample that was used in the PCR reaction was plotted per participant against the average Cq-value of the 6 amoeba samples (Supplemental Fig. S2, Supplemental Digital Content, http://links.lww.com/ICO/B549). This graph reveals that there was no correlation between the percentage of the original sample used in the PCR reaction and the performance of the analysis of these amoeba samples.

Figure 3 shows for each participant whether a pretreatment was used in the procedure for the amoeba samples, and if so, which one(s). Square symbols are used for participants who reported not using any form of pretreatment, and circles are used for the participants who used some form of pretreatment. Inspection of the results of the analyses of the cyst samples indicates that using some form of pretreatment improves the overall performance of these samples, as 6 of the 7 participants who scored a Cq-value below the median use a pretreatment procedure and had no false negative results (Fig. 3C). Consequently, 5 of the 6 laboratories that did not use a pretreatment procedure reported an average Cq-value above the median value for the set of cyst samples. Furthermore, 3 of those 5 participants with an average Cq-value above the median and not using a pretreatment reported a false negative result for one of these cyst samples (Fig. 3C). For the set of trophozoite samples, the difference in performance, whether a pretreatment was part of the analytical procedure, was less extreme. Three of the 7 participants who scored for the set of trophozoite samples, an average Cq-value below the median, did not use any pretreatment (Fig. 3D). Furthermore, for the set of cyst samples, all 5 participants who reported the lowest Cq-values used some form of pretreatment, whereas for the trophozoite samples, 2 of the 5 laboratories that reported the lowest Cq-values did not use any pretreatment and even scored the second and third best Cq-value (Fig. 3D).

## DISCUSSION

Early diagnosis of *Acanthamoeba* keratitis is critical, as this leads to more favorable outcomes compared with late diagnosis.^4^ Therefore, an easily implemented, reliable, and sensitive diagnostic tool is needed, aspects in which NAAT are potentially superior to all other currently used diagnostic methods. However, the exact procedures that are used for NAAT-based detection of *Acanthamoeba* spp. differ substantially between laboratories. By participating in an EQAS, laboratories can compare results and identify methodologies that might affect their diagnostic performance.

Overall, we found a clear correlation between the number of *Acanthamoeba* cysts or trophozoites in the samples and the detection rate. In the samples containing 20 cysts or trophozoites, the detection rate was considerably lower than in the samples containing 200 cysts or trophozoites (Fig. 2). Although the exact numbers of *Acanthamoeba* cysts and/or trophozoites present in clinical samples are unknown, it is assumed that only a few amoebae are retrieved in cornea scrapings from patients, indicating the importance of successfully detecting low numbers of amoebae.

The ultimate goal for *Acanthamoeba* diagnostics is an excellent qualitative performance, that is, a high detection rate. However, the qualitative performance is related to the quantitative one, and therefore, the quantitative performance also deserves attention. In case of samples with only a few amoebae, laboratories with relatively high Cq-values for the cyst and trophozoite samples run a higher risk of false negative results than laboratories with a more efficient analysis of amoeba samples.

The relative quantitative performances in this EQAS for the sets of DNA, cyst, and trophozoite samples were determined by sorting the participants based on the average of their reported Cq-values for these sets (Fig. 3). This revealed, as could be expected, that the variation in the efficiency to detect amoeba DNA is greater for the cyst and trophozoite samples, which have to be subjected to the entire procedure of pretreatment, DNA extraction, and amplification than for the samples that contain already isolated amoeba DNA and only need DNA amplification by PCR. Comparing the efficiencies (average Cq-values) of the analyses of the sets of samples by the various participants revealed that laboratories that reported low Cq-values for cysts tended to also report low Cq-values for trophozoites (Fig. 3C,D). However, comparing per participant the results for the set of DNA samples with the results for the 2 sets of amoeba samples shows that laboratories with a high efficiency (low Cq-value) for the DNA samples not automatically also scored well (low Cq-value) in the amoeba samples (Fig. 3A, B).

One of the limitations of this EQAS is that no clinical samples were used but that laboratory-grown cultures were distributed. This could have influenced the results, as corneal scrapings contain other components than the culture medium. However, obtaining homogeneous and sufficient volumes of representative clinical specimens (cornea scrapings) is impossible for *Acanthamoeba*. In other words, any EQAS needs to make use of cultured material. Another limitation is that only one *Acanthamoeba* strain was used in this EQAS. However, as all used molecular targets were aimed at conserved regions of the *Acanthamoeba* genome, it is unlikely that using multiple strains will change the results of the participating laboratories. Although an EQAS is a valuable tool to analyze and compare the relative performance of diagnostic laboratories, an EQAS on the NAAT diagnostics of *Acanthamoeba* is not the most ideal way to decide what the best pretreatment procedure is for clinical samples. The number of participants and variations in the pretreatment procedures, and especially the various possible combinations thereof, are usually limited. The conclusive identification of pretreatment procedures that result in a reliable detection of *Acanthamoeba* in a routine diagnostic setting awaits further systematic studies. Still, based on our findings, some general remarks on the pros and cons of the various pretreatment procedures can now be made.

The use of any pretreatment in an *Acanthamoeba* NAAT is meant to improve the lysis of the cysts and trophozoites, resulting in the release of the intracellular DNA, which can then be amplified in the subsequent PCR step. The observation that none of the 6 laboratories that use proteinaseK in their pretreatment procedure, reported a false negative result for the 6 amoeba samples, indicates that the use of proteinase K is a helpful addition for a reliable procedure (Fig. 3A). It is unknown whether a pretreatment consisting only of proteinase K would lead to similar results, as all 6 of these laboratories used another method in addition to proteinase K in the pretreatment; heat–shock, bead-beating, or a freeze–thaw cycle.

The use of a pretreatment procedure can pose the risk that the extra handling steps that are involved in the pretreatment procedure result in extra technical or logistic hurdles, leading to inadequate results. In that respect, the addition of proteinase K, the use of a heat–shock, or a freeze–thaw cycle seem not too challenging. Bead-beating, in all its variants, a fierce physical homogenization method, seems to be a good choice for pretreatment in *Acanthamoeba* diagnostics because the cysts are very robust. On the other hand, in contrast to the other 3 methods, any bead-beating variant necessarily involves an extra transfer of the sample and dilution of the usually small samples. The laboratory with the most efficient analysis of the cyst samples used proteinase K and bead beating (Fig. 3C). However, the other laboratory that used bead beating (but without the use of proteinase K) reported 2 false negatives for the 3 samples containing cysts (Fig. 3C). Bead beating was also used by the 2 laboratories that were not included in the quantitative evaluation, as they reported inhibition or negative results for all amoeba samples.

As mentioned earlier, this EQAS cannot be used and was not meant to identify the ultimate method for the diagnosis of *Acanthamoeba* keratitis. This study was developed to demonstrate that the development of an EQAS for the NAAT-based detection of *Acanthamoeba* cysts and trophozoites benefits clinical laboratories, as they can compare their methods and results with those of others, which can provide suggestions for improvements.

## Supplementary Material
