# Supplementary material for: An International External Quality Assessment Scheme to Assess the Diagnostic Performance of Polymerase Chain Reaction Detection of Acanthamoeba Keratitis
Source: Cornea. 2023 May 4;42(8):1027–33. doi: 10.1097/ICO.0000000000003275 (PMC10306335; doi:10.1097/ICO.0000000000003275)
Supplement: Supplementary file 1 [file cornea-42-1027-s001.docx]

**Supplemental TABLE S1.** Uniformity of the samples was verified by an expert laboratory

by a five-fold determination of the Cq-value of the amoeba samples

| Sample type | 20  cysts | 200  cysts | 2000  cysts | 20 trophozoites | 200 trophozoites | 2000 trophozoites |
| --- | --- | --- | --- | --- | --- | --- |
| Replicate 1 | 32.2 | 29.0 | 26.4 | 32.4 | 29.7 | 25.6 |
| Replicate 2 | 28.2 | 27.8 | 25.0 | 32.3 | 29.4 | 26.7 |
| Replicate 3 | 28.8 | 28.8 | 24.9 | 32.5 | 29.1 | 24.1 |
| Replicate 4 | 30.6 | 28.3 | 25.1 | 34.0 | 29.1 | 26.5 |
| Replicate 5 | 30.3 | 28.8 | 24.6 | 33.5 | 29.1 | 25.4 |
| **Average** | 30.0 | 28.5 | 25.2 | 32.9 | 29.3 | 25.7 |
| **Standard deviation** | 1.4 | 0.4 | 0.6 | 0.7 | 0.2 | 0.9 |
