# Supplementary material for: An International External Quality Assessment Scheme to Assess the Diagnostic Performance of Polymerase Chain Reaction Detection of Acanthamoeba Keratitis
Source: Cornea. 2023 May 4;42(8):1027–33. doi: 10.1097/ICO.0000000000003275 (PMC10306335; doi:10.1097/ICO.0000000000003275)
Supplement: Supplementary file 2 [file cornea-42-1027-s002.docx]

**Supplemental TABLE S2.** The stability of the samples was examined by two expert laboratories by comparison of the Cq-value at the time of distribution (Cq start)

with the Cq-value 4 months later (Cq 4 mo)

| Sample | Expert lab 1 | | | Expert lab 2 | | | Average Δ^c^ |
| --- | --- | --- | --- | --- | --- | --- | --- |
|  | Cq start | Cq 4 mo | Δ^a^ | Cq start | Cq 4 mo | Δ^a^ | of labs 1 and 2 |
| 20 cysts | 32.6 | 32.0 | *0.6* | 31.3 | 31.1 | *0.2* | *0.40* |
| 200 cysts | 30.5 | 30.0 | *0.5* | 27.9 | 28.8 | *-0.9* | *-0.20* |
| 2000 cysts | 27.8 | 27.4 | *0.4* | 24.5 | 27.2 | *-2.7* | *-1.15* |
|  | | | | | | | |
| 20 trophozoites | 30.5 | 30.0 | *0.5* | 34.0 | 33.5 | *0.5* | *0.50* |
| 200 trophozoites | 27.1 | 26.7 | *0.4* | 30.7 | 30.6 | *0.1* | *0.25* |
| 2000 trophozoites | 24.9 | 24.4 | *0.5* | 28.2 | 25.8 | *2.4* | *1.45* |
|  | | | | | | | |
| Low [DNA] | 34.7 | 33.4 | *1.3* | 34.5 | 34.7 | *-0.2* | *0.55* |
| Medium [DNA] | 31.1 | 30.2 | *0.9* | 30.7 | 31.7 | *-1.0* | *-0.05* |
| High [DNA] | 27.7 | 27.5 | *0.2* | 28.0 | 28.2 | *-0.2* | *0* |
|  |  |  |  |  |  |  | *0.19* |
|  |  | Average Δ^b^ | *0.59* |  | Average Δ^b^ | *-0.20* |  |

^a^ the difference between the Cq-value at the time of distribution and 4 months later (Δ = Cq start minus Cq after 4 months)

^b^ the average of the averages per laboratory

^c^ the average difference of the two laboratories per sample
