# Supplementary material for: An International External Quality Assessment Scheme to Assess the Diagnostic Performance of Polymerase Chain Reaction Detection of Acanthamoeba Keratitis
Source: Cornea. 2023 May 4;42(8):1027–33. doi: 10.1097/ICO.0000000000003275 (PMC10306335; doi:10.1097/ICO.0000000000003275)
Supplement: Supplementary file 3 [file cornea-42-1027-s003.pdf]

Supplemental Table S3 Reported Cq-values and calculations used for the ordering of the laboratories in the various sample types.

A The reported Cq-values

Participants:	Low [DNA]	Medium [DNA]	High [DNA]		2 cysts	20 cysts	200 cysts	2000 cysts	2 trophozoites	20 trophozoites	200 trophozoites	2000 trophozoites	
A	37.0	33.0	29.0		negative	33.0	31.0	27.0	37.0	33.0	28.0	25.0	
B	34.8	31.5	27.5		37.0	negative	31.6	28.5	negative	35.5	34.3	28.6	
C	32.0	28.0	25.5		negative	30.0	33.5	30.0	negative	35.0	35.0	28.0	
D	34.7	31.1	27.7		negative	32.6	30.5	27.8	36.0	30.5	27.1	24.9	
E	35.4	33.0	29.6		negative	negative	negative	32.6	negative	37.9	36.3	32.6	
F	34.5	30.7	28.0		32.4	31.3	27.9	24.5	34.5	34.0	30.7	28.2	
G	36.4	31.7	28.6		37.8	35.0	35.7	30.7	38.7	38.9	31.7	26.4	
H	35.3	31.3	28.3		negative	negative	31.9	28.7	negative	negative	33.7	27.9	
I	35.5	31.4	28.3		inhibited	inhibited	inhibited	inhibited	inhibited	inhibited	inhibited	inhibited	All amoeba samples inhibited - Participant not included in sorting
J	33.1	30.1	26.9		negative	negative	negative	negative	negative	negative	negative	negative	All amoeba samples negative - Participant not included in sorting
K	38.3	35.1	31.1		41.4	39.5	34.5	32.2	negative	38.5	35.4	30.1	
L	32.5	29.3	25.9		negative	32.0	28.9	25.9	negative	32.5	32.5	26.2	
M	33.6	31.1	28.0		negative	negative	31.2	28.4	37.3	32.0	30.8	24.6	
N	34.3	32.2	28.6		37.5	34.1	28.9	26.4	negative	negative	34.2	29.7	
O	36.6	33.3	29.9		negative	33.9	34.3	31.2	40.3	37.6	36.1	29.0	
P	32.9	29.5	26.0		37.5	33.2	29.0	25.6	inhibited	38.0	31.9	22.8	
Average	34.8	31.4	28.1		37.3	33.5	31.5	28.5	37.3	35.3	32.7	27.4	
Median	34.8	31.4	28.2		37.5	33.1	31.2	28.5	37.2	35.3	33.1	28.0	
SD	1.7	1.7	1.5		2.6	2.4	2.4	2.4	1.9	2.8	2.7	2.5	
				positive	6/16=37.5 %	10/16 = 62,5 %	13/16 = 81,25 %	14/16 = 87,5 %	6/16=37.5 %	12/16 = 75 %	14/16 = 87,5 %	14/16 = 87,5 %	

Participants:	Pre-treatment	
A	-	BB = Bead-beating
B	-	FT = Freeze-thaw cycle
C	PK, FT	HS = Heat-shock
D	PK HS	PK = proteinase K
E	BB	?? = classified
F	PK BB	— no pre-treatment
G	-	
H	-	
I	PK BB	
J	BB	
K	PK HS	
L	PK HS	
M	-	
N	??	
O	-	
P	PK FT	

B Cq-values used for sorting

Participants:	20 cysts	200 cysts	2000 cysts	20 trophozoites	200 trophozoites	2000 trophozoites	Number of incorrect results in these 6 samples: False negatives	inhibited	Average 3 cysts + 3 trophozoites	Participant
A	33.0	31.0	27.0	33.0	28.0	25.0	0	0	29.50	A
B	39.5	31.6	28.5	35.5	34.3	28.6	1	0	33.00	B
C	30.0	33.5	30.0	35.0	35.0	28.0	0	0	31.92	C
D	32.6	30.5	27.8	30.5	27.1	24.9	0	0	28.90	D
E	39.5	39.5	32.6	37.9	36.3	32.6	2	0	36.40	E
F	31.3	27.9	24.5	34.0	30.7	28.2	0	0	29.43	F
G	35.0	35.7	30.7	38.9	31.7	26.4	0	0	33.07	G
H	39.5	31.9	28.7	39.5	33.7	27.9	2	0	33.53	H
I							0	6		I
J							6	0		J
K	39.5	34.5	32.2	38.5	35.4	30.1	0	0	35.03	K
L	32.0	28.9	25.9	32.5	32.5	26.2	0	0	29.67	L
M	39.5	31.2	28.4	32.0	30.8	24.6	1	0	31.08	M
N	34.1	28.9	26.4	39.5	34.2	29.7	1	0	32.13	N
O	33.9	34.3	31.2	37.6	36.1	29.0	0	0	33.68	O
P	33.2	29.0	25.6	38.0	31.9	22.8	0	0	30.08	P

Participants:	Pre-treatment	Average of 3 DNA Cq's	Average of 3 cyst Cq's	Average of 3 trophozoite Cq's
A	-	33.0	30.3	28.7
B	-	31.3	33.2	32.8
C	PK, FT	28.5	31.2	32.7
D	PK HS	31.2	30.3	27.5
E	BB	32.7	37.2	35.6
F	PK BB	31.1	27.9	31.0
G	-	32.2	33.8	32.3
H	-	31.6	33.4	33.7
I	PK BB	31.7		
J	BB	30.0		
K	PK HS	34.8	35.4	34.7
L	PK HS	29.2	28.9	30.4
M	-	30.9	33.0	29.1
N	??	31.7	29.8	34.5
O	-	33.3	33.1	34.2
P	PK FT	29.5	29.3	30.9

Participants:	Average of 3 DNA Cq's	Average of 3 cyst Cq's	Average of 3 trophozoite Cq's
A	33.0	30.3	28.7
B	31.3	33.2	32.8
C	28.5	31.2	32.7
D	31.2	30.3	27.5
E	32.7	37.2	35.6
F	31.1	27.9	31.0
G	32.2	33.8	32.3
H	31.6	33.4	33.7
I	31.7		
J	30.0		
K	34.8	35.4	34.7
L	29.2	28.9	30.4
M	30.9	33.0	29.1
N	31.7	29.8	34.5
O	33.3	33.1	34.2
P	29.5	29.3	30.9

C Data for Figure 3A

Sorting: Ascending order in the average of 3 cyst + 3 trophozoite Cq-values

Average 3 cyst + 3 trophozoite	Participant	Pre-treatment	Order
28.90	D	PK HS	1
29.43	F	PK BB	2
29.50	A	-	3
29.67	L	PK HS	4
30.08	P	PK FT	5
31.08	M	-	6
31.92	C	PK, FT	7
32.13	N	??	8
33.00	B	-	9
33.07	G	-	10
33.53	H	-	11
33.68	O	-	12
35.03	K	PK HS	13
36.40	E	BB	14
--	I	PK BB	
--	J	BB	

E Calculation of spreading around median Cq-value

	Average of 3 DNA Cq's	Average of 3 cyst Cq's	Average of 3 trophozoite Cq's
	28.50	27.90	27.50
	29.23	28.93	28.67
	29.47	29.27	29.13
	30.03	29.80	30.40
	30.90	30.30	30.90
	31.07	30.33	30.97
	31.17	31.17	32.33
	31.27	33.03	32.67
	31.63	33.13	32.80
	31.70	33.20	33.70
	31.73	33.37	34.23
	32.23	33.80	34.47
	32.67	35.40	34.67
	33.00	37.20	35.60
	33.27		
	34.83		
median	31.45	32.10	32.50
median - 1	30.45	31.10	31.50
median + 1	32.45	33.10	33.50
count	8	2	3
	8/16 = 50%	2/14 = 14%	3/14 = 21%

D Participants ordered for cysts, throphozoites and DNA Cq-values using the average of Cq-values of cysts + trophozoites (20; 200; 2000):

Data for Figure 3C

Participants:	Pre-treatment	Order in 3C + 3T	Average of 3 cyst Cq's
F	PK BB	2	27.9
L	PK HS	4	28.9
P	PK FT	5	29.3
N	??	8	29.8
D	PK HS	1	30.3
A	-	3	30.3
C	PK FT	7	31.2
M	-	6	33.0
O	-	12	33.1
B	-	9	33.2
H	-	11	33.4
G	-	10	33.8
K	PK HS	13	35.4
E	BB	14	37.2
		median	32.1
		average	31.9
		delta max - min	9.3

Data for Figure 3D

Participants:	Pre-treatment	Order in 3C + 3T	Average of 3 trophozoite Cq's
D	PK HS	1	27.5
A	-	3	28.7
M	-	6	29.1
L	PK HS	4	30.4
P	PK FT	5	30.9
F	PK BB	2	31.0
G	-	10	32.3
C	PK FT	7	32.7
B	-	9	32.8
H	-	11	33.7
O	-	12	34.2
N	??	8	34.5
K	PK HS	13	34.7
E	BB	14	35.6
		median	32.5
		average	32.0
		delta max - min	8.1

Data for Figure 3B

Participants	Pre-treatment	Order in 3C + 3T	Average of 3 DNA Cq's
C	PK FT	7	28.5
L	PK HS	4	29.2
P	PK FT	5	29.5
J	BB	X	30.0
M	-	6	30.9
F	PK BB	2	31.1
D	PK HS	1	31.2
B	-	9	31.3
H	-	11	31.6
N	??	8	31.7
I	PK BB	X	31.7
G	-	10	32.2
E	BB	14	32.7
A	-	3	33.0
O	-	12	33.3
K	PK HS	13	34.8
		median	31.5
		average	31.4
		delta max - min	6.3
