# Supplementary figures and images for: An International External Quality Assessment Scheme to Assess the Diagnostic Performance of Polymerase Chain Reaction Detection of Acanthamoeba Keratitis
Source: Cornea. 2023 May 4;42(8):1027–33. doi: 10.1097/ICO.0000000000003275 (PMC10306335; doi:10.1097/ICO.0000000000003275)

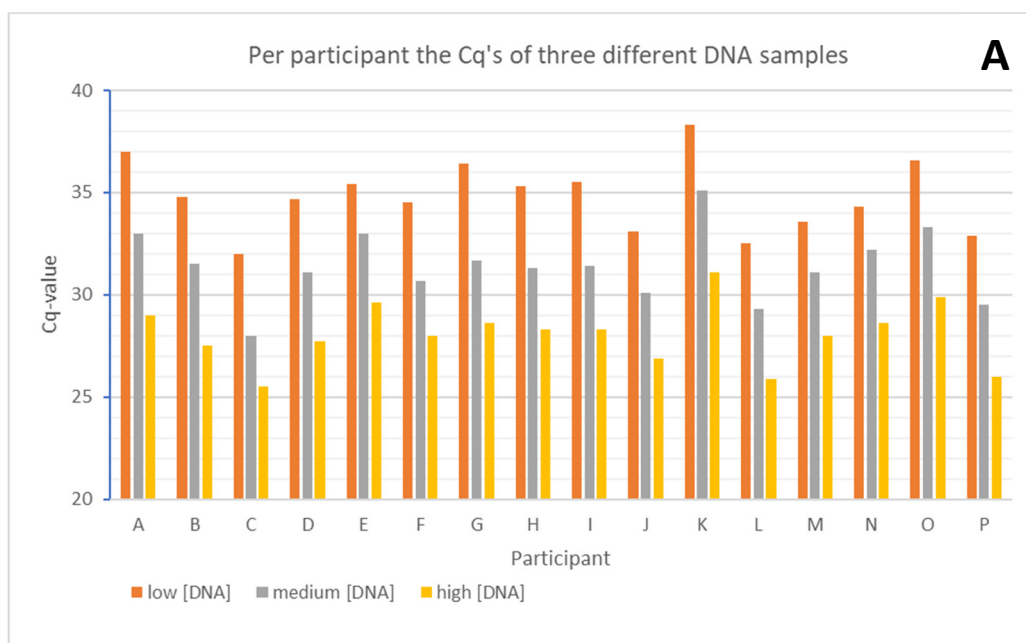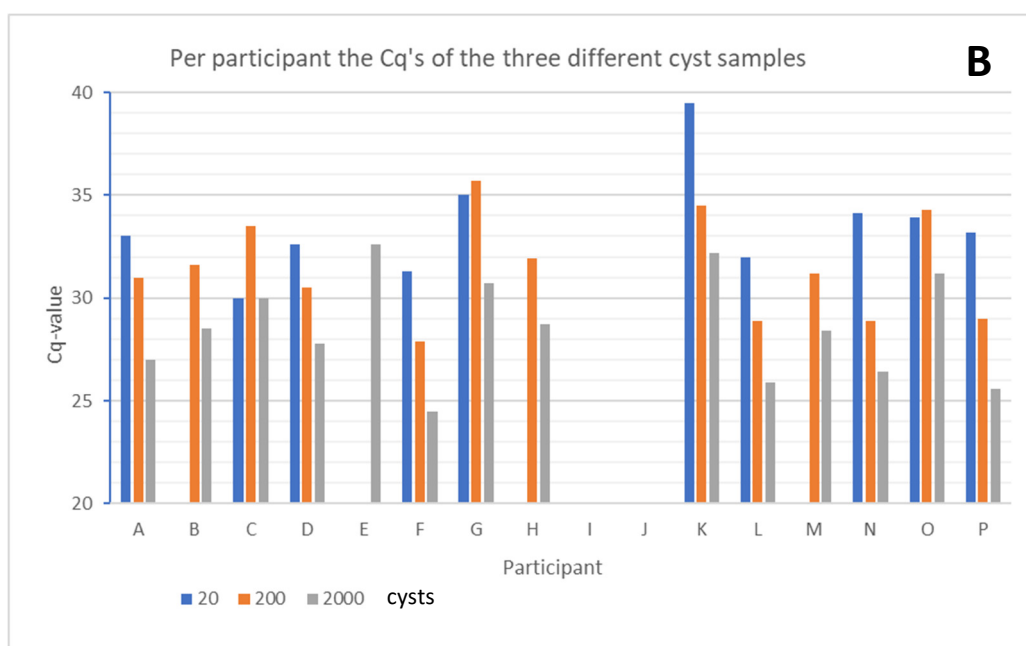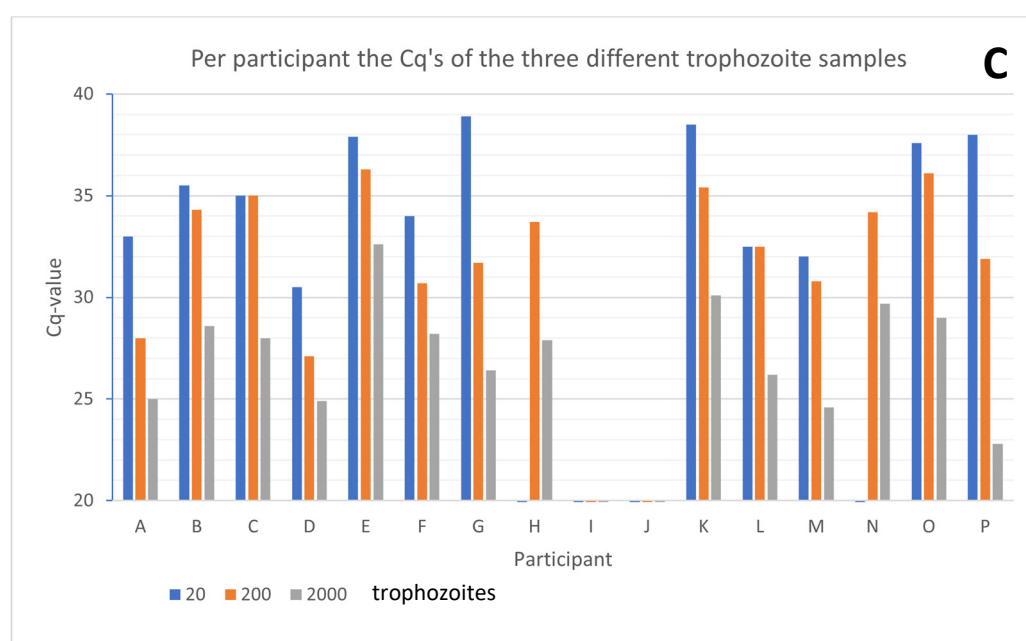

Supplemental Figure S1

Supplement: Supplementary file 4 [file cornea-42-1027-s004.pdf]

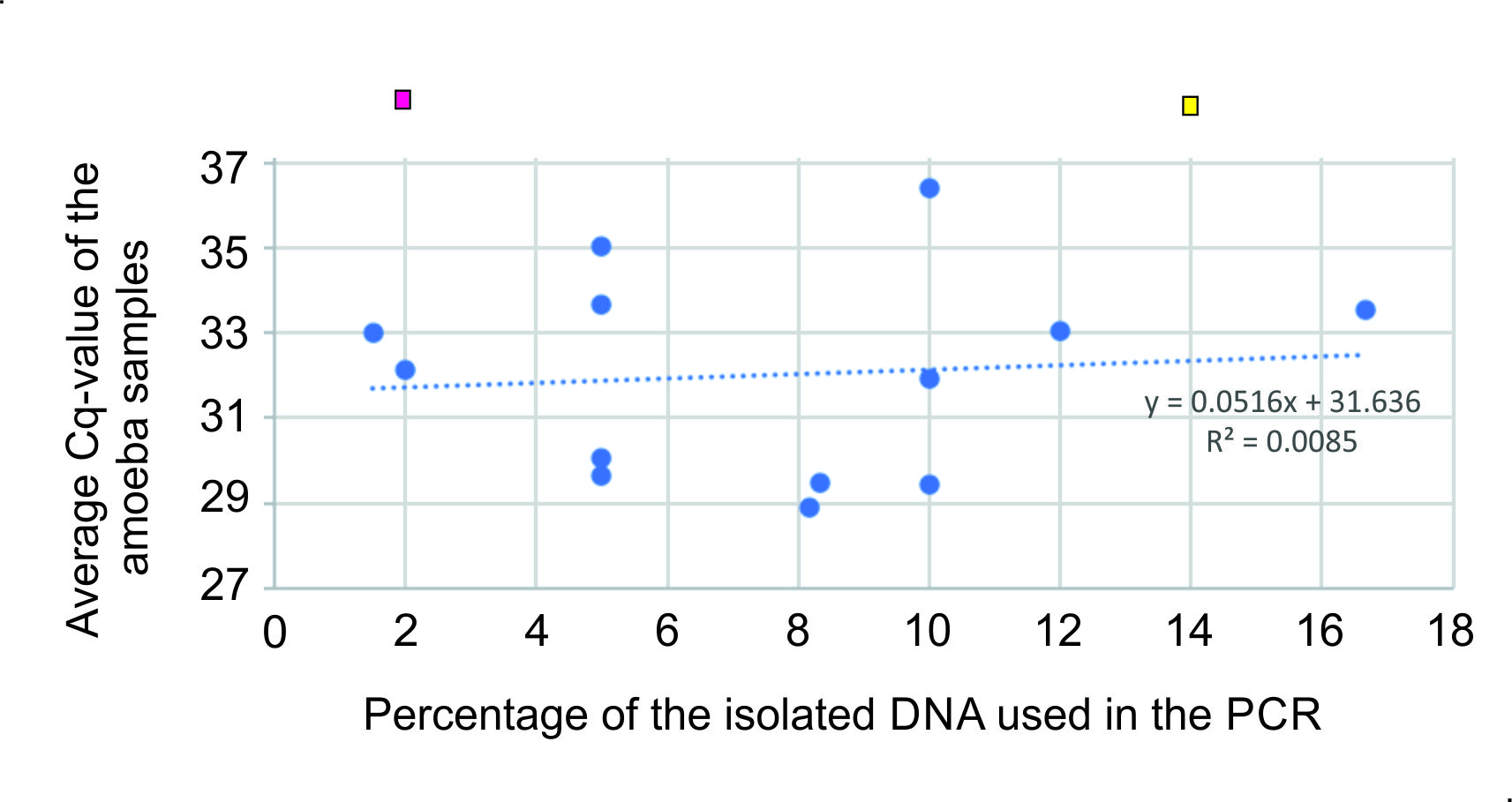

Supplement: Supplementary file 5 [file cornea-42-1027-s005.jpg]
